# Supplementary material for: Translation and natural selection of micropeptides from long non-canonical RNAs
Source: Nat Commun. 2022 Oct 31;13:6515. doi: 10.1038/s41467-022-34094-y (PMC9622821; doi:10.1038/s41467-022-34094-y)
Supplement: Supplementary file 1 — Supplementary Information [file 41467_2022_34094_MOESM1_ESM.pdf]

Supplementary Figure 1

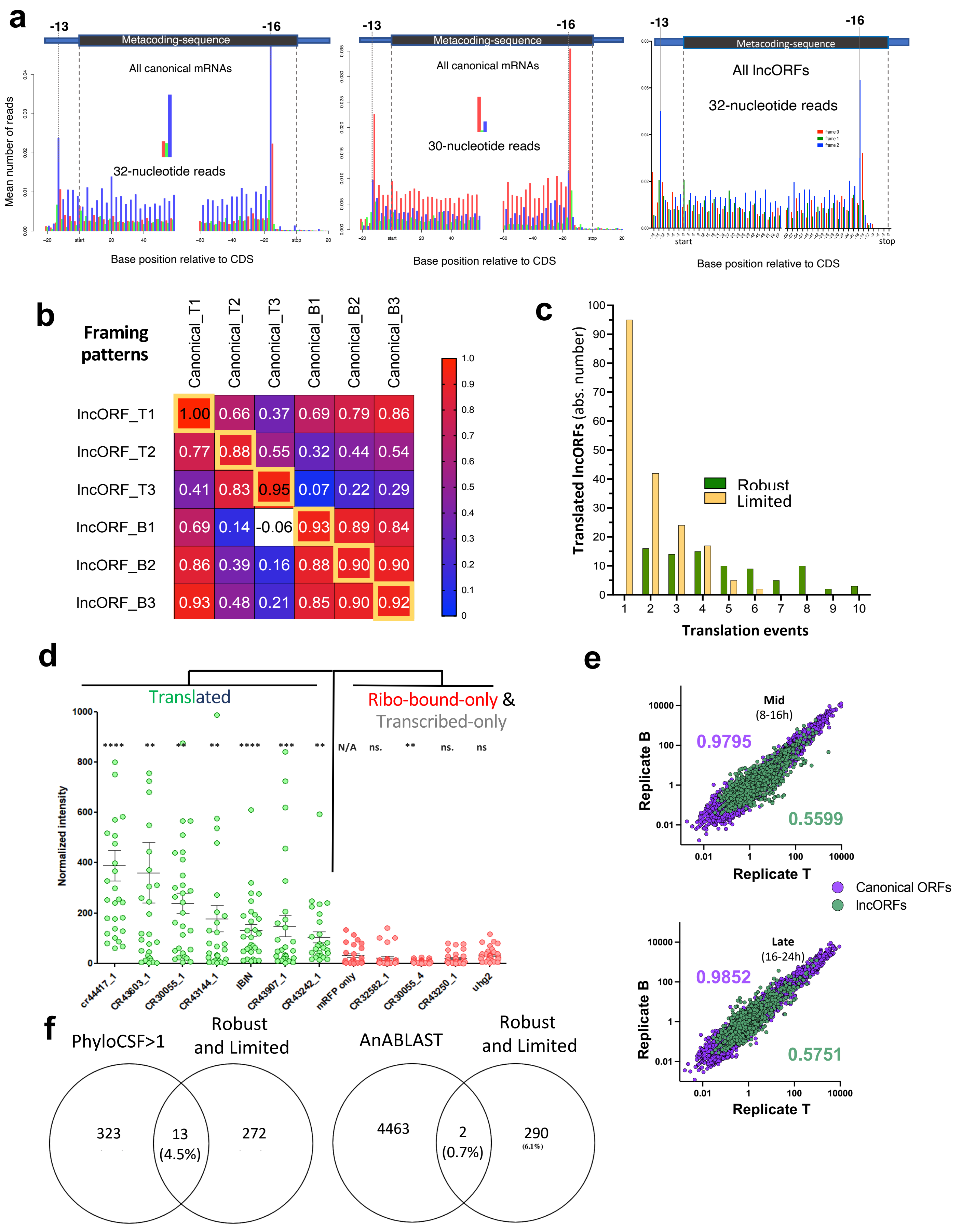

Supplementary Figure 1 | a) Riboseq framing coverage. Metagene plots for 32 (left) and 30 (middle) nucleotide-long RPF alignments to positions -20 to +60 and -60 to +20 around AUG and STOP codons, respectively, for all canonical ORFs, or all lncORFs (right, 32 nt. RPFs) **b) Correlation heatmap for lncORF and canonical framing**, per embryo Ribo-Seq replicate. Framing patterns per RPF length **(**26-36 nt.) were used for both lncORFs and canonical ORF classes. Heatmap colours correspond to Spearman's *rho* for pairwise comparisons. Yellow squares: correlation in framing across classes, for the same biological replicate. T: 'T' replicate. B: 'B' replicate. *e.g.* T1: Replicate T, 0-8h. **c) Total number of translation events across *Drosophila melanogaster* embryogenesis**, for all lncORFs detected as having limited (yellow) or robust (green) translation. **d) Quantification of tagged lncORF expression** levels for translated (green) and not translated (red) lncORFs. N=30 independent cells for each of the constructs tested. "*" indicates p<0.05; "**" indicates p<0.01; "***" indicates p<0.001; "****" indicates p<0.0001. Exact *p*-values: CR44417_1*: p*=0.00009; CR43907_1*: p*=0.0074; CR43144_1*: p*=0.0061; CR43603_1*: p*=0.0081; CR30055_1*: p*=0.00009; IBIN*: p*=0.0001; CR43242_1*: p*=0.0023; CR32582_1*: p*=0.4154; Uhg2*: p*=0.5488; CR30055_4*: p*=0.0066; CR43250_1*: p*=0.2898; Unpaired *t*-tests, two-tailed, pairwise comparisons between each construct and mRFP control. **e)** Correlation or Embryo Ribo-Seq RPKM values. Plot and Spearman's *rho* values for lncORF (green) and canonical ORFs (purple) $RPKM^{FP}$ values across replicates B (*y* axis) and T (*x* axis) during mid (8-16h) and late (16-24h)-embryogenesis. **f) PhyloCSF and AnABLAST codingness predictions** have very limited overlap (4.45% and 0.7%, respectively) with our set of translated lncORFs (either Robust or Limited) as determined by Ribo-seq.

Supplementary Figure 2

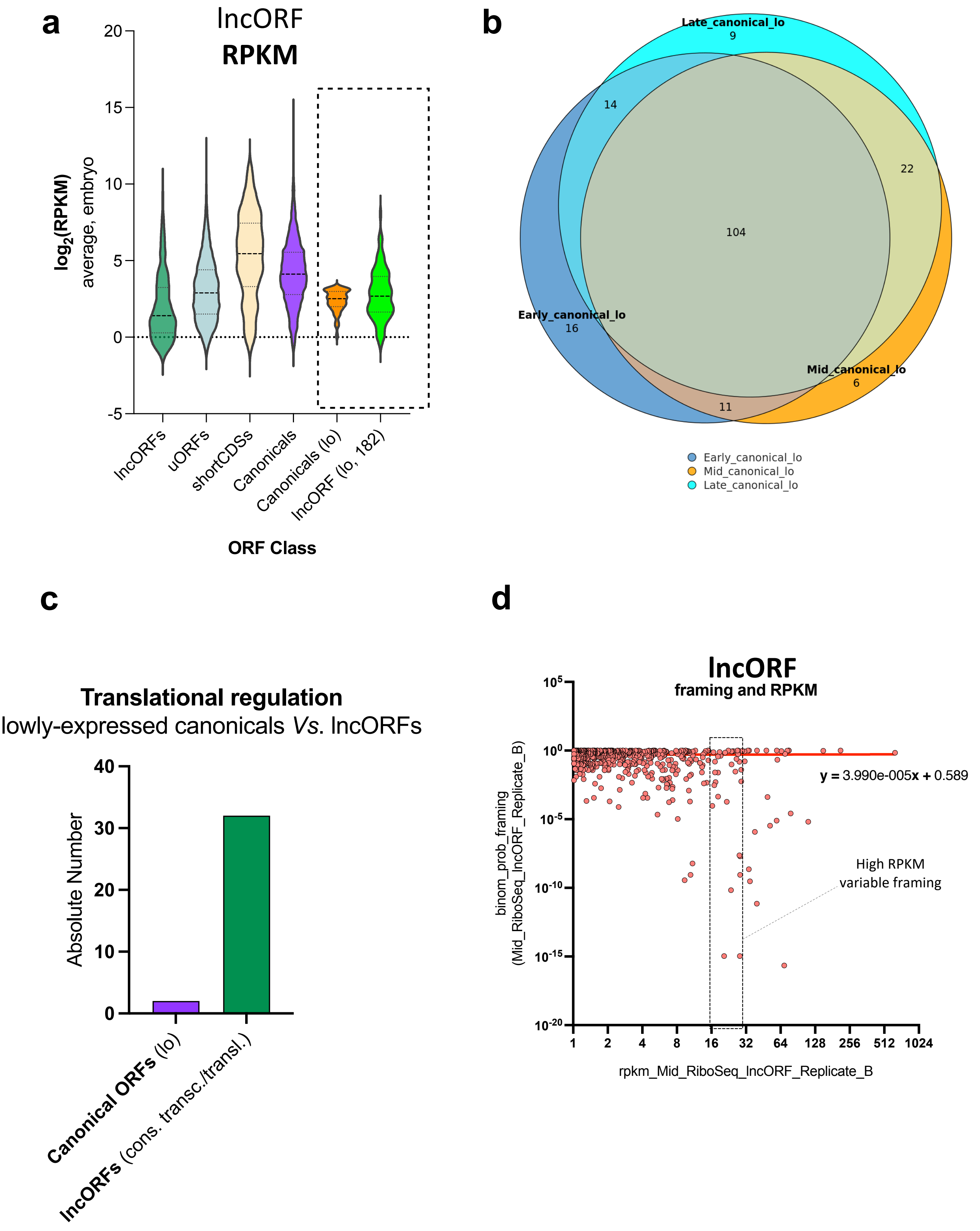

Supplementary Figure 2 | a) RPKM value distributions for matched populations of canonical and lncORFs. To control for artefactual translational regulation across stages due to low lncORF expression, we selected an equivalent number of translated canonical ORFs matching the lower RPKM range of constitutively transcribed lncORFs (Dotted line). Other ORF classes are represented for comparison. **b) Qualitative translational regulation of lowly-expressed canonical ORFs across embryogenesis.** Venn diagram depicting the detection of translation across embryonic stages for constitutively-transcribed embryonic canonical ORFs (annotated, > 100AA; N=182). **c) Quantitative translational regulation for lowly expressed ORFs across embryogenesis.** Absolute number of canonical ORFs and lncORFs displaying significant modulations in TE (translational efficiency) across embryonic stages (Z-ratio Early-Mid or Mid-Late > |1.5|). **d) relationship between lncORF RPKM and framing in the embryo.** X-Y plot showing RPKM (X) and binomial probability (Y) for every lncORF in Mid-embryogenesis (8-16h), replicate B. Linear regression denoted by red line, with corresponding equation below. Dotted square serves to illustrate a number of lncORFs with similarly high RPKM values (between 16 and 32) and a wide range of framing patterns, from aperiodic (most, top) to triplet periodicity (below).

Supplementary Figure 3

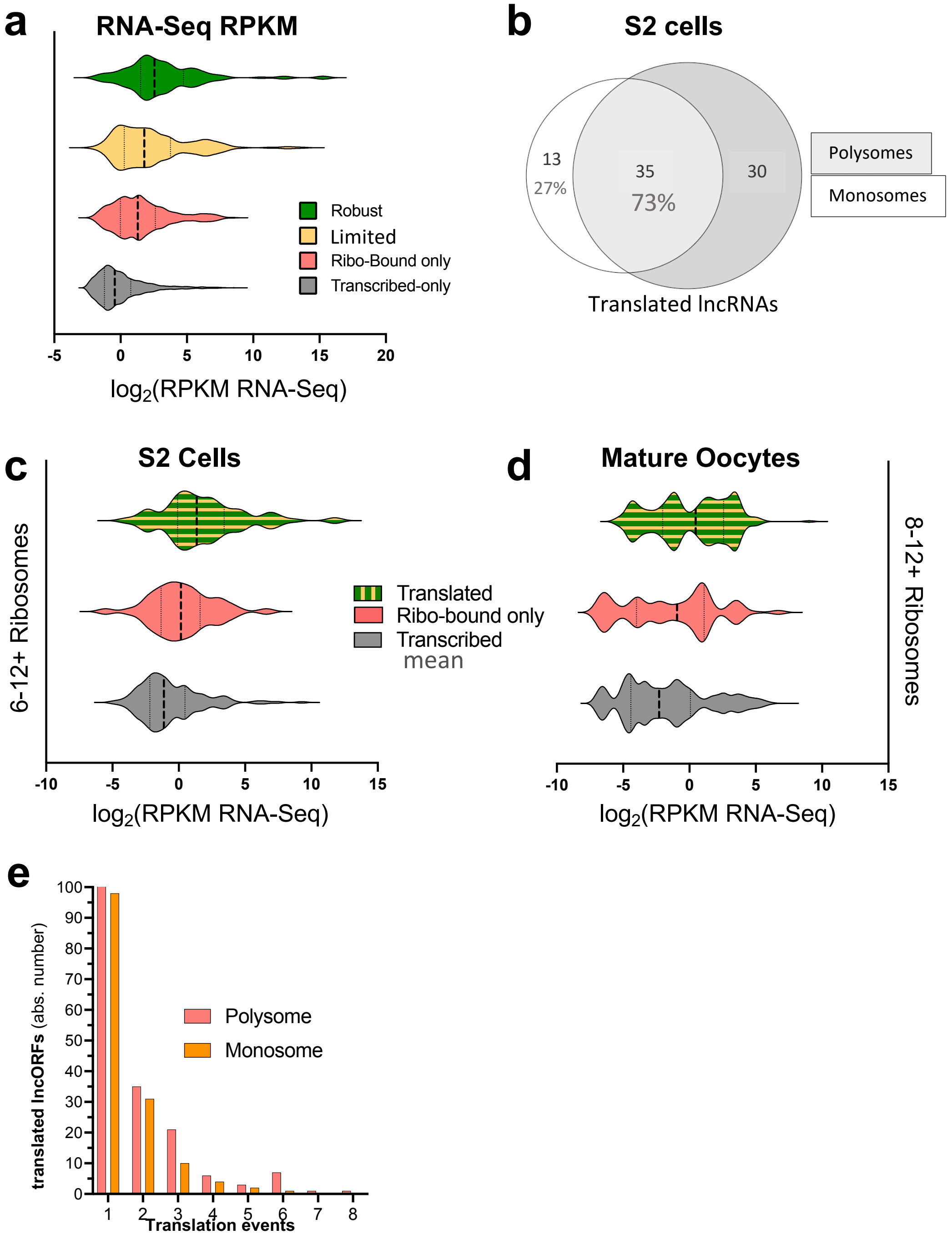

Supplementary Figure 3 | a) RNA-Seq RPKM across lncORF translation classes. Violin plots of log_2-transformed RNA-Seq RPKM (average across embryogenesis) for all lncORFs in each translation class. Thick dotted lines denote median, thin dotted lines denote lower and upper quartiles. **b) Number of translated lncRNAs in S2 cells.** Proportional Venn diagram depicting lncRNAs containing translated ORFs in either monosomes (white circle) or polysomes (grey circle). Absolute numbers in black, percentages of total below (grey). **c) Loading of translated lncRNAs in S2 cell polysomes**. Violin plots of RPKM values for lncRNAs detected by polysomal profiling in the 6-12 ribosome fraction of S2 cells. **d) Loading of translated lncRNAs in mature oocytes**. Violin plots of RPKM values for lncRNAs detected by polysomal profiling in the 8-12 ribosome fraction of mature oocytes from *Drosophila melanogaster*. **e) lncORF translation events in monosomes and polysomes of S2 cells. Histogram of absolute number of** lncORFs and detected number of translation events ($p<0.01$ per lncORF per RPF length, in lengths 26-36) in either monosomes or polysomes.

Supplementary Figure 4

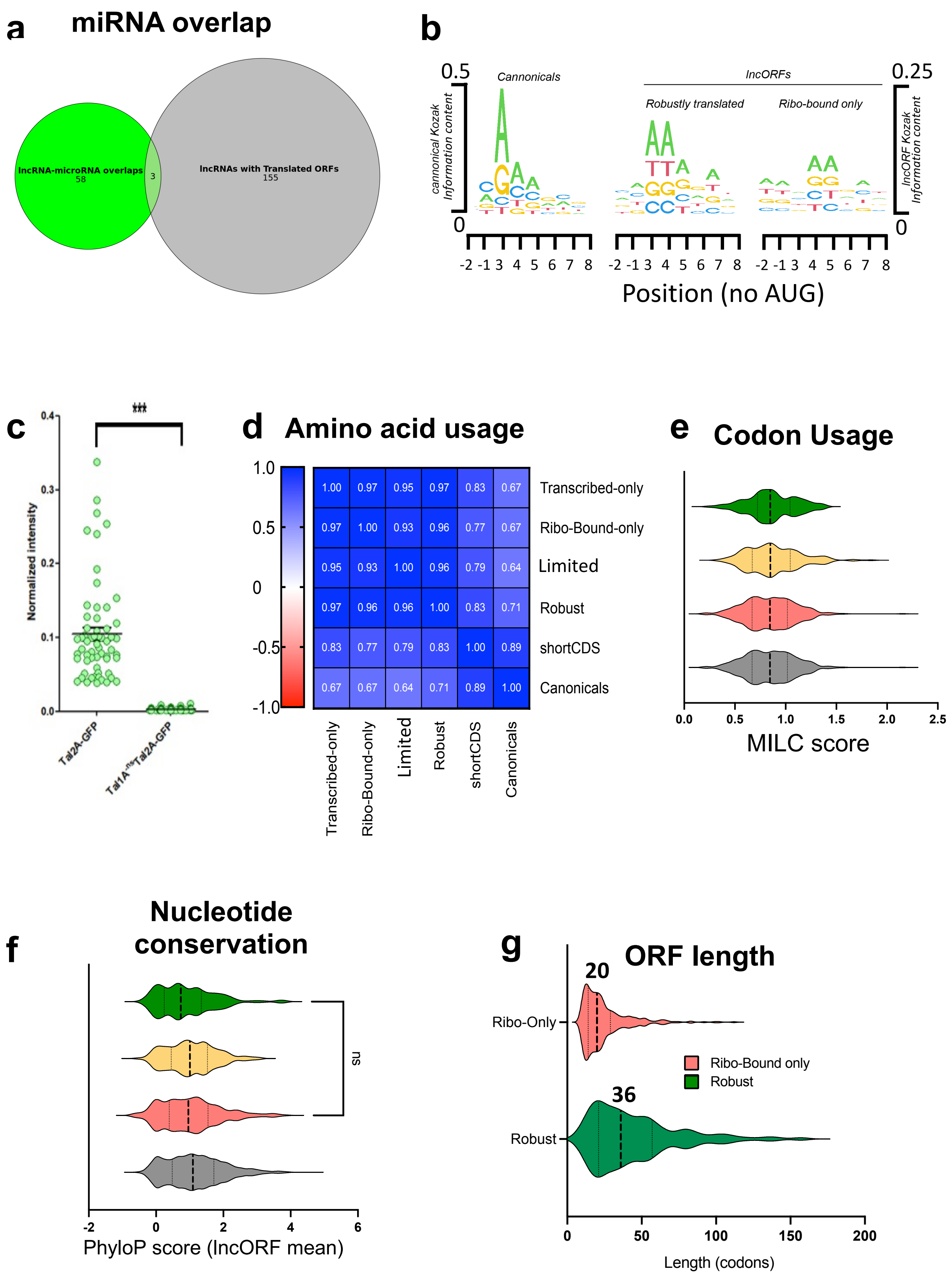

Supplementary Figure 4 a) Comparison between lncRNA translation and microRNA production in *cis*. lncRNAs with translated ORFs were compared with those serving as hosts for microRNAs, with the two groups showing minimal overlap. **b) Sequence logos of Kozak contexts** reflecting the better scores obtained by translated lncORFs compared to ribo-bound ony lncORFs. Notice also the qualitative differences between these classes; ribo-bound lncORFs lack the highly represented adenine in position 3, whereas Robustly translated lncORFs show a clear over representation of adenines in that position. **c) Quantification of translation from *tarsal-less* constructs.** Each green dot represents the quantification of an individual cell expressing GFP-tagged ORF 2A expression from *tarsal-less* constructs with (left) or without (right) a STOP codon. N=30 independent cells for both conditions. Asterisks (**) denote *p*-value = 0.0008 (Unpaired t-test, one-tailed). **d) Correlation heatmap of amino acid usages across ORF classes in the *D. melanogaster* transcriptome**. Numbers denote *Pearson's r* values for each pairwise correlations. Transcribed-only, Ribo-bound only, Limited and Robust denote lncORF classes. **e) Codon optimality across lncORF categories.** Violin plots of codon usage, as compared to canonical ORFs using the MILC score (Measure Independent of Length and Composition). Thick dotted lines denote median, thin dotted lines denote lower and upper quartiles. **f) Nucleotide-level conservation of lncORF classes.** Violin plots for average PhyloP scores across the ORF, for distinct lncORF translation classes, using pre-computed genome-wide scores for multiple alignments of 26 genomes to the *D. melanogaster* genome. *p*=02878; Mann-Whitney test, two-tailed. ***g*) Robustly translated lncORFS tend to be larger than lncORFs that are Ribo-bound only.** Thick dotted lines denote median, thin dotted lines denote lower and upper quartiles.

Supplementary Figure 5

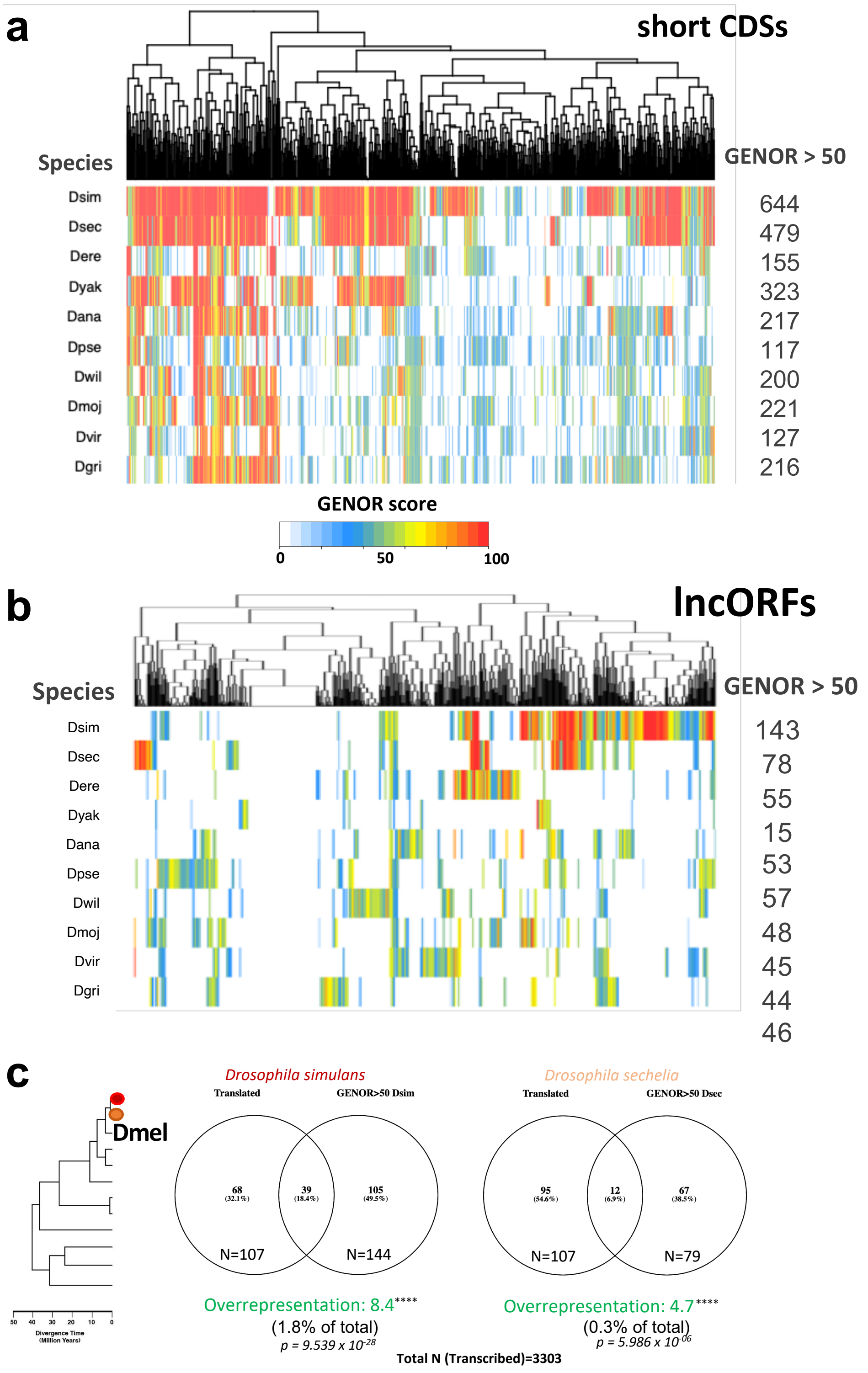

d

ORF_ID	start	chr	strand	length (codons)	RiboSeq rpkm average	Independent framing calls	Kozak Score	Dsim GENOR score	dN/dS Dsim
FBtr0340272_1	33	2R	+	41	23.2778196	14	1.1986	72.682927	Inf
FBtr0081385_1	60	2L	+	21	8.6312575	8	2.9544	70.47619	0.431742
FBtr0086842_1	22	2R	+	21	18.4485596	12	3.9778	93.809524	0.52062
FBtr0339421_2	78	2R	-	61	4.79481231	5	2.8675	66.065574	0.461379
FBtr0340282_1	23	2R	+	28	10.7719149	11	2.8633	51.428571	0.62681
FBtr0340526_3	86	2R	+	51	8.56682554	2	-1.4808	53.72549	0.414885

Supplementary Figure 5 | GENOR heatmaps for consevation scores across the *Drosphoila sp.* phylogeny for **a)** all annotated smORFs (shortCDSs) and **b)** all lncORFs with ribosome binding. Warmer colours denote higher conservation score (red: 100% conservation), whereas colder colours denote lower conservation scores (white: no conservation). Column on the right of each heatmap denotes number of *D. melanogaster* ORFs with detected homologues (GENOR score higher than 50) in each Drosophilid species. **c)** statistical overrepresentation of robustly translated lncORFs in the conserved lncORF group, as defined by GENOR scores higher than 50 in *Drosophila simulans (Dsim*, left panel, $p = 9.539 \times 10^{-28}$) and *Drosophila sechelia* (*Dsec,* right panel, $p = 5.986 \times 10^{-06}$). Green: Representation factor statistic (see Methods). Red: Species placing in the phylogeny. **d**) Individual examples of conserved, robustly translated lncORFs in *Drosophila simulans*, selected from the 39 overlapping lncORFs in panel S5c, left. *p-values* correspond to representation factor analysis, see Methods.

Supplementary Table 1

Supplementary Table 1: Sequencing read numbers for all samples used

Embryo

Protocol	Replicate	Stage	RAW READS Total Sample	RAW READS TOTAL	ORF-ALIGNED READS Total Sample	ORF-ALIGNED READS TOTAL
Poly-Ribo-Seq	T	Early	730,411,918	2,047,810,976	80,543,642<br>11.03%	570,558,097
	B	Early	322,959,034		100,025,645<br>30.97%	
	T	Mid	356,182,680		113,774,297<br>31.94%	
	B	Mid	170,312,394		40,998,164<br>24.07%	
	T	Late	309,593,498		179,207,862<br>57.88%	
	B	Late	158,351,452		56,008,487<br>35.37%	

Protocol	Replicate	Stage	RAW READS		GENOME-ALIGNED READS	
RNA-Seq	T	Early	108,457,666	604,933,230	39,350,875<br>36.28%	265,136,599
	B	Early	99,986,496		73,969,154<br>73.98%	
	T	Mid	54,333,403		13,534,556<br>24.91%	
	B	Mid	170,312,394		67,655,830<br>39.72%	
	T	Late	68,857,596		19,879,597<br>28.87%	
	B	Late	102,985,675		50,746,587<br>49.28%	

S2 Cells

			RAW READS	GENOME-ALIGNED READS
Poly-Ribo-Seq	S2	Monosomes	71,338,560	12420079<br>17.41%
	S2	Polysomes	67,920,154	35113316<br>51.70%
RNA-Seq	S2	Total RNA	82,358,730	69684819<br>84.61%

Supplementary Table 1 | Sequencing used in this study. Number of reads per sample analysed, for both embryonic samples (top) or S2-cell samples (bottom). Both initial ("raw") and ORF-aligned reads are depicted. In the case of RNA-Seq, genome-aligned reads are reported. Red numbers denote percentage of ORF-aligned reads, with respect to the initial value, per sample.
