# Supplementary material for: Translation and natural selection of micropeptides from long non-canonical RNAs
Source: Nat Commun. 2022 Oct 31;13:6515. doi: 10.1038/s41467-022-34094-y (PMC9622821; doi:10.1038/s41467-022-34094-y)
Supplement: Supplementary file 3 — Description of Additional Supplementary Files [file 41467_2022_34094_MOESM3_ESM.pdf]

Description of Additional Supplementary Files

File Name: Supplementary Data 1
Description: This supplementary data file consists of an Excel table (XLSX format) containing relevant information on the all the lncRNA ORFs considered in this study including their reference name, transcript and gene of origin, genomic coordinates, evolutionary patterns as measured by GENOR and dN/dS and expression levels in both RNA-Seq and Ribo-Seq RPKM per replicate.

File Name: Supplementary Data 2
Description: This supplementary data file consists of an Excel table (XLSX format) containing substitution pattern information relevant for the measurement of dN/dS in all the lncRNA ORFs considered in this study, for pariwise alignments between *Drosophila melanogaster* and either *Drosophila simulans* (Dsim) or *Drosophila sechellia* (Dsec).
