# Supplementary material for: Translation and natural selection of micropeptides from long non-canonical RNAs
Source: Nat Commun. 2022 Oct 31;13:6515. doi: 10.1038/s41467-022-34094-y (PMC9622821; doi:10.1038/s41467-022-34094-y)
Supplement: Supplementary file 6 — Reporting Summary [file 41467_2022_34094_MOESM6_ESM.pdf]

nature portfolio

Corresponding author(s): Juan Pablo Couso

Last updated by author(s): Sep 29, 2022

Reporting Summary

Nature Portfolio wishes to improve the reproducibility of the work that we publish. This form provides structure for consistency and transparency in reporting. For further information on Nature Portfolio policies, see our Editorial Policies and the Editorial Policy Checklist.

Statistics

For all statistical analyses, confirm that the following items are present in the figure legend, table legend, main text, or Methods section.

n/a	Confirmed	
☐	☒	The exact sample size (*n*) for each experimental group/condition, given as a discrete number and unit of measurement
☐	☒	A statement on whether measurements were taken from distinct samples or whether the same sample was measured repeatedly
☐	☒	The statistical test(s) used AND whether they are one- or two-sided<br>*Only common tests should be described solely by name; describe more complex techniques in the Methods section.*
☐	☒	A description of all covariates tested
☐	☒	A description of any assumptions or corrections, such as tests of normality and adjustment for multiple comparisons
☐	☒	A full description of the statistical parameters including central tendency (e.g. means) or other basic estimates (e.g. regression coefficient) AND variation (e.g. standard deviation) or associated estimates of uncertainty (e.g. confidence intervals)
☐	☒	For null hypothesis testing, the test statistic (e.g. *F*, *t*, *r*) with confidence intervals, effect sizes, degrees of freedom and *P* value noted<br>*Give P values as exact values whenever suitable.*
☐	☒	For Bayesian analysis, information on the choice of priors and Markov chain Monte Carlo settings
☐	☒	For hierarchical and complex designs, identification of the appropriate level for tests and full reporting of outcomes
☐	☒	Estimates of effect sizes (e.g. Cohen's *d*, Pearson's *r*), indicating how they were calculated

Our web collection on statistics for biologists contains articles on many of the points above.

Software and code

Policy information about availability of computer code

Data collection	No software was used for data collection.
Data analysis	For S2 cell lncORF expression analysis we used standard Image J plugins (v 1.53c). GraphPad Prism (v.9.1.1) and R (v.4.0.3) were used for statistical analyses. R packages coRdon (v.1.8.0), riboSeqR (v.1.0.0) and ape (v.5.6-2) were used for nucelotide and amino acid sequence analyses. EMBOSS (v.6.6.0.0) was used to predict ORFs. bedtools (v.2.30.0) was used for analyses involving genomic coordinates. A novel software, GENOR, has been used for homology detection and is available. The source code for GENOR is available at https://github.com/CouLab/GENOR.

For manuscripts utilizing custom algorithms or software that are central to the research but not yet described in published literature, software must be made available to editors and reviewers. We strongly encourage code deposition in a community repository (e.g. GitHub). See the Nature Portfolio guidelines for submitting code & software for further information.

Data

Policy information about availability of data

All manuscripts must include a data availability statement. This statement should provide the following information, where applicable:

- Accession codes, unique identifiers, or web links for publicly available datasets
- A description of any restrictions on data availability
- For clinical datasets or third party data, please ensure that the statement adheres to our policy

All sequecing data used in this manuscript is available in https://www.ncbi.nlm.nih.gov/geo/, under accession number: GSE204739. Further data and materials are available under request to the corresponding author.

Field-specific reporting

Please select the one below that is the best fit for your research. If you are not sure, read the appropriate sections before making your selection.

☒ Life sciences ☐ Behavioural & social sciences ☐ Ecological, evolutionary & environmental sciences

For a reference copy of the document with all sections, see nature.com/documents/nr-reporting-summary-flat.pdf

Life sciences study design

All studies must disclose on these points even when the disclosure is negative.

Sample size	Sequencing depth was determined following the results of Patraquim et al. 2020, Gen. Biol. Specifically, sequencing depth (number of reads) was increased until statistically significant differences (by binomial cumulative tests) could be obtained from two biological replicas when measuring tri-nucleotide periodicity in the 5´mapping of the reads. Our sample size of duplicates per Ribo-seq and RNA-seq experiments, particularly considering the sequencing depth obtained in this study, goes beyond the current standards applied to these methods. The numbers chosen for Flag expression quantification (at least 30 cells per condition) were enough to obtain significant differences as evaluated by standard one tailed t test, with acceptable standard deviations.
Data exclusions	No data was excluded, however we depleted bochemically and computationally, prior to analysis, the sequences belonging to ribosomal and tRNAs.
Replication	All ribosomal profiling and RNA-seq data presented here was obtained from experiments performed in duplicates, likewise for experimental validation in S2 cells. All attempts at replication were successful
Randomization	No specific experimental variations (apart from developmental) were tested in this study. The raw observations (RNA sequencing reads) are generated automatically by sequencing machines and come effectively randomized, as a mixture from thousands of cells from hundreds of embryos.
Blinding	No specific experimental variations (apart from developmental) or hypothesis were tested in the original Ribosomal profiling collection. The raw observations (RNA sequencing reads) are descriptive in nature and generated automatically by sequencing machines from external service providers, who only had access to our identifier codes. Thus, the raw data was obtained blindly without experimenter input or interpretation.

Reporting for specific materials, systems and methods

We require information from authors about some types of materials, experimental systems and methods used in many studies. Here, indicate whether each material, system or method listed is relevant to your study. If you are not sure if a list item applies to your research, read the appropriate section before selecting a response.

Materials & experimental systems

n/a	Involved in the study	
☐	☒	Antibodies
☐	☒	Eukaryotic cell lines
☒	☐	Palaeontology and archaeology
☐	☒	Animals and other organisms
☒	☐	Human research participants
☒	☐	Clinical data
☒	☐	Dual use research of concern

Methods

n/a	Involved in the study	
☒	☐	ChIP-seq
☒	☐	Flow cytometry
☒	☐	MRI-based neuroimaging

Antibodies

Antibodies used	We used commercial anti-FLAG (Sigma, F3165, clone M2) and anti-mouse -FITC (jackson, 715-545-150) antibodies.
Validation	Both commercial antibodies have been validated for use in Drosophila melanogaster in Aspden, J. L. et al. Extensive translation of small Open Reading Frames revealed by Poly-Ribo-Seq eLife 3, e03528 (2014).

Eukaryotic cell lines

Policy information about cell lines

Cell line source(s)	Drosophila S2 cells were obtained from the Drosophila Genome Resource Centre

Authentication	These cells have been thoroughly authenticated by the DGRC. No specific authentification, other than morphological, was carried out for these cells.
Mycoplasma contamination	No mycoplasma contamination was detected in our cells.
Commonly misidentified lines (See ICLAC register)	No commonly misidentified cell lines were used in this study

Animals and other organisms

Policy information about studies involving animals; ARRIVE guidelines recommended for reporting animal research

Laboratory animals	The experiments described in his study were performed in Oregon Red strain (established in or before 1925: Genetic Variations of Drosophila melanogaster, Lindsley and Grell, 1972, Carnegie Inst of Washington, Washington, D.C., U.S.A.) of Drosophila melanogaster, also known as fruitfly. Flies of both sexes, from 1 to 20 days old were used indiscriminately.
Wild animals	No wild animals were used in the study
Field-collected samples	No field collected samples were used in the study
Ethics oversight	This study did not require an ethical approval

Note that full information on the approval of the study protocol must also be provided in the manuscript.
